# Chemical Composition, In Vitro Antimicrobial, Free-Radical-Scavenging and Antioxidant Activities of the Essential Oil of *Leucas inflata* Benth

**DOI:** 10.3390/molecules22030367

**Published:** 2017-02-27

**Authors:** Ramzi A. Mothana, Omar M. Noman, Ebtesam S. Al-Sheddi, Jamal M. Khaled, Mansour S. Al-Said, Adnan J. Al-Rehaily

**Affiliations:** 1 Department of Pharmacognosy, College of Pharmacy, King Saud University, P.O. Box 2457, Riyadh 11451, Saudi Arabia; onoman@ksu.edu.sa (O.M.N.); ealsheddi@ksu.edu.sa (E.S.A.-S.); msalsaid@ksu.edu.sa (M.S.A.-S.); ajalreha@ksu.edu.sa (A.J.A.-R.); 2 Department of Pharmacognosy, Faculty of Pharmacy, Sana’a-University, P.O. Box 33039, Sana’a, Yemen; 3 Department of Botany and Microbiology, College of Science, King Saud University, Riyadh 11451, Saudi Arabia; gkhaled@ksu.esu.sa

**Keywords:** *Leucas inflata*, GC, GC/MS, essential oil, antimicrobial, antioxidant

## Abstract

The essential oil of *Leucas inflata* Balf.f. (Lamiaceae), collected in Yemen, was analyzed using gas chromatography (GC) and gas chromatography-mass spectrometry (GC-MS) techniques. Forty-three components were recognized, representing 89.2% of the total oil. The *L. inflata* volatile oil was found to contain a high percentage of aliphatic acids (51.1%). Hexadecanoic acid (32.8%) and *n*-dodecanoic acid (7.8%) were identified as the major compounds. Oxygenated monoterpenes were distinguished as the second significant group of constituents (16.0%). Camphor (6.1%) and linalool (3.2%) were found to be the main components among the oxygenated monoterpenes. In addition, the volatile oil was assessed for its antimicrobial activity against four bacterial strains and one yeast species using broth micro-dilution assay for minimum inhibitory concentrations (MIC). In addition, antioxidant activity was measured utilizing the anti-radical activity of the sable free radical 2,2-diphenyl-1-picrylhydrazyl (DPPH) and β-Carotene-linoleic acid assays. The oil of *L. inflata* showed an excellent antibacterial activity against only the tested Gram-positive bacteria with a MIC-value of 0.81 mg/mL. Furthermore, the oil demonstrated, at a concentration of 1 mg/mL, a weak to moderate antiradical and antioxidant activity of 38% and 32%, respectively.

## 1. Introduction

The genus *Leucas* (family: Lamiaceae) is represented by over 80 members, which are broadly distributed throughout Asia, Africa, and India [1]. Members of this genus have been widely used as remedies in folk medicine in Africa and India for many diseases, and, thus, they have received considerable attention. Species, e.g., *L. lavandulaefolia*, have been described to have hepatoprotective, antipyretic, anti-diarrheal, hypoglycemic, antitussive, and wound healing activities [2,3,4,5,6,7]. Moreover, *L. aspera* was found to have antimicrobial, antiinflammatory, antinociceptive, antioxidant, and cytotoxic activities [8,9,10]. *L. martinicensis* showed antibacterial, anthelmintic, and antimalarial activities [11,12,13].

The genus *Leucas* is represented in Yemen by five species, including *Leucas inflata* Benth., which grows as a perennial undershrub, and is widespread and frequent on open rocky plains and stony hills on the high plateau extending through the plains around the Sana’a governorate [14]. The plant is used for the treatment of kidney diseases and toothaches by native people in Yemen. Previous works on *L. inflata* roots have demonstrated that the extracts have anti-inflammatory and analgesic potential [15,16]. Evaluating the accessible current literature, no report was found relating to chemical or pharmacological investigations of the volatile oil of the aerial part of *L. inflata*. In a continuation of our studies on natural volatile oils of species from our region, and their conceivable pharmacological activities, we report here, for the first time, the chemical composition and antimicrobial and antioxidant activities of the volatile oil of *Leucas inflata*.

## 2. Results and Discussion

### 2.1. Chemical Composition of the Volatile Oil

The volatile oil obtained via hydrodistillation of the flowering aerial part of *L. inflata* was colorless and aromatic in a yield of 0.3% (*w*/*w* dry plant material). The chemical composition of *L. inflata* essential oil is shown in Table 1, where the compounds are listed in the order of their elution on the CP-Sil 5 CB column. Moreover, retention index, percentage, and identification tool of each compound are demonstrated in Table 1. A total of 43 components, representing 89.2% of the total essential oil, were identified. It is significant to note that no reports have been found relating to gas chromatography-mass spectrometry (GC-MS) analysis of the *L. inflata* volatile oil. Moreover, this work presents the first study on the antibacterial and antiradical qualities of the sable free radical 2,2-diphenyl-1-picrylhydrazyl (DPPH) and the antioxidant activities of the tested essential oil. *L. inflata* volatile oil was identified by a high content of aliphatic acids (51.1%). Among the aliphatic acids, hexadecanoic acid (32.8%) and *n*-dodecanoic acid (7.8%) were found to be the main constituents (Table 1). Oxygenated monoterpenes were identified as the second major group of compounds (16.0%). Camphor (6.1%) and linalool (3.2%) were detected as the main compounds among oxygenated monoterpenes (Table 1).

Comparing our results with previously published data on the chemical composition of volatile oils of the *Leucas* species revealed some important quantitative and qualitative differences between them. The GC-MS analysis revealed that our *Leucas inflata* was mainly differentiated by its aliphatic acids content (Table 1).

In former studies, the chemical composition of the volatile oils of various *Leucas* species, e.g., *L. virigata*, *L. aspera*, *L. indica*, *L. glabrata*, *L. milanjiana* and *L. deflexa*, were investigated [17,18,19,20,21,22]. We recently reported the chemical content of the volatile oil of the Soqotraen *L. virgate*, which was distinguished by a high composition of oxygenated monoterpenes (50.8%). Among the oxygenated monoterpenes, camphor (20.5%) was found to be the basic component [20]. While chemical investigation of the volatile oil of the African *L. aspera* detected carvone, carvacrol, and menthol as major components [17], a recent report by Joshi et al. (2016) [18] on the volatile oil of the Indian *L. aspera* uncovered that the oil is rich in sesquiterpene hydrocarbons (47.7%), where β-caryophyllene was the fundamental constituent with a percentage of 34.2% [18]. Whereas Joshi (2014) [23] confirmed the dominance of β-caryophyllene (51.1%) in the *L. indica* essential oil, Vagionas et al. (2007) [22] found that the fundamental chemical class in *L. glabrata* is oxygenated monoterpenes (64.4%), with menthone (31.8%) and pulegone (11.4%) as essential constituents. Moreover, high contents of β-cubebene (38.1%), α-pinene (19.7%), and trans-caryophyllene were detected in *L. milanjiana* oil [19]. Additionally, the investigation performed on the volatile oil of the *L. deflexa* leaves revealed a high amount of sesquiterpene hydrocarbons (80.3%) with germacrene-d (27.8%), β-caryophyllene (24.1%) and α-humulene (9.9%) as major components [21].

These results indicate that seasonal and interannual changes in climate, as well as environmental and geographical factors, are definitely involved in the creation of a different and noticeable chemical composition of *L. inflata* volatile oil. Hexadecanoic acid may, therefore, serve as a chemical marker to differentiate *L. inflata* from other related species of the genus *Leucas*.

### 2.2. Antimicrobial Activity

Generally, the tested Gram-positive bacteria were more sensitive to the volatile oil than Gram-negative bacteria. No antifungal activity was seen against *Candida albicans*. The essential oil of *L. inflata* showed a powerful antibacterial effect with a minimal inhibitory concentration (MIC-value) of 0.81 mg/mL against *Bacillus subtilis* and *Staphylococcus aureus* (Table 2).

The predomination of aliphatic acids, such as hexadecanoic acid, could explain the obtained powerful antimicrobial effect [24]. Moreover, oxygenated monoterpenoids like camphor and linalool were also described to be accountable for the antimicrobial activity of various essential oils [25,26]. The possible synergistic actions of these components in the essential oil ought to be granted consideration. Our results are in a good agreement with studies of volatile oils and methanol extracts prepared from other *Leucas* members [8,17,22,27].

### 2.3. Antioxidant Activity

In this study, the possible antioxidant effects of the *L. inflata* volatile oil were evaluated using two methods, namely 2,2-diphenyl-1-picrylhydrazyl- (DPPH) and β-Carotene-linoleic acid assays. The volatile oil showed only moderate anti-radical activity of the stable free radical DPPH (Table 3). Thus, the volatile oil, at a concentration of 1 mg/mL, exhibited a low to moderate anti-radical activity of DPPH (38%) compared with the high anti-radical activity of ascorbic acid (96%). In addition, Table 3 demonstrates the mean antioxidant activity, based on the β-carotene bleaching rate of the essential oil. The observed antioxidant activity was also weak (32%) in comparison to that of rutin (91%).

This observed activity is definitely connected with the low content of phenolic components, e.g., thymol and carvacrol, in the investigated oil [28]. In contrast to a previous study, the methanol extract of *L. inflata* exhibited a high antioxidant activity (80%) at 1 mg/mL [29]. This remarkable result could be ascribed to the total polyphenol and total flavonoid contents of the methanol extract of *L. inflata* [29]. In addition, remarkable antioxidant activities were also reported for methanol and ethanol extracts of various *Leucas* members, such as *L. mollissima* and *L. aspera* [10,30].

## 3. Materials and Methods

### 3.1. Plant Materials

The aerial parts of *L. inflata* were collected from Sana’a governorate in April 2008. The plant was identified in the Department of Pharmacognosy, Faculty of Pharmacy, Sana’a University. A voucher specimen was deposited in the Department of Pharmacognosy, Faculty of Pharmacy, Sana’a University.

### 3.2. Preparation of the Essential Oil

The oil was obtained from the dry and ground, aerial flowering plant material of *L. inflata* using hydrodistillation (3 h), using a Clevenger-type apparatus. Then, the volatile oil was dried by anhydrous sodium sulfate and stored at 5 °C until further analysis and investigation.

### 3.3. Gas Chromatography Analysis

The gas chromatographic investigation was carried out using a Hewlett Packard GC (5890 Series II, Hewlett-Packard Company, Wilmington, DE, USA) with a Flame Ionization Detector (FID). A fused silica capillary column (CP-Sil 5 CB column, 30 m × 0.25 mm i.d., film thickness 0.26 μm, Agilent Technologies, Santa Clara, CA, USA) was used as a stationary phase. As a carrier gas, nitrogen was utilized at a flow rate of 0.7 mL/min. The temperatures of the injector and the detector were set at 250 °C and 280 °C, respectively. The oven temperature was kept at 45 °C, and then slowly increased to 280 °C at 3 °C/min, and finally held isothermally for 22 min. A volume of 1.0 μL of the essential oil (1/100 diluted in pentane, *v*/*v*) was injected (split mode, split ratio 1:16). Calculation of the percentage of each peak area was done on basis of the FID signal, utilizing the GC HP-Chemstation program (Agilent Technologies, Santa Clara, CA, USA).

### 3.4. Gas Chromatography-Mass Spectrometry

The GC–MS investigation was performed utilizing a gas chromatograph connected with a VG Analytical 70-250S mass spectrometer (VG Analytical Ltd., Manchester, UK). A fused silica capillary column (CP-Sil 5 CB, 25 m × 0.25 mm i.d., film thickness 0.4 μm, from Chromback, Varian) was the used stationary phase. As a carrier gas, helium was utilized at a flow rate of 1 mL/min. The temperature of the injector was 200 °C. The oven temperature was raised from 80 °C to 270 °C at 10 °C/min, and finally held isothermally for 20 min. An electron impact mode of 70 eV was utilized for the detection with a mass scan range from 35 to 600 amu.

### 3.5. Identification of Components

Identification of individual constituents was done by comparing the RI-values and mass spectra with data generated under similar conditions using a special two-dimensional search algorithm software with an integrated library, considering the RI and MS similarities [31]. Additionally, MassLib (1996–2008) was applied with other available libraries like Wiley Registry (4th ed.), NIST/EPA/NIH Mass spectral Library (2005) and the Adams Library [32]. Furthermore, available authentic reference compounds were used in the comparison. The determination of RI was attained based on a homologous *n*-alkanes series (C8–C30) under identical working conditions.

### 3.6. Determination of Antimicrobial Activity

The antimicrobial activity of the volatile oil was evaluated using the broth microdilution protocol to determine MICs (minimal inhibitory concentrations) [33]. The microorganisms were acquired from the Bernhard-Nocht Institute (BNI) for Tropical Medicine, Hamburg, Germany. Test microorganisms included four bacteria (*Staphylococcus aureus* BNI 18, *Bacillus subtilis* BNI 28, *Escherichia coli* BNI 2, and *Pseudomonas aeruginosa* BNI 20) and one yeast (*Candida albicans* BNI 33). For the test, 96-well microdilution plates were utilized. In this protocol, duplicate two-fold serial dilutions of the essential oil (100 μL/well) were prepared in nutrient broth containing 5% (*v*/*v*) dimethyl sulphoxide. Wells in columns 1–8 were adequate to test eight dilutions of the oil, which was indispensable for an endpoint minimal inhibition concentration (MIC). One hundred microliters of microbial cell suspensions (10^5^ CFU/mL) were prepared in the appropriate broth (nutrient broth for bacteria or tryptone soya yeast extract broth for *Candida*) and was added to all cells of the 96-well plates except for columns 9, 10, 11, and 12, which were used for saline, essential oil, media sterility, and growth controls, respectively. Standard antibiotics, such as amoxicillin, gentamicin, and nystatin, were utilized as positive controls in duplicate. The 96-well plates were incubated at 37 ± 1 °C for 19 h; then, microbial growth was observed. To show the growth of bacteria more clearly, the plates were treated with a solution of a p-iodonitro-tetrazolium violet (Sigma-Aldrich, St. Louis, MO, USA) (40 µL of 0.2 mg/mL in each well) and the plates were incubated at 37 ± 1 °C for 30 min. The pink color indicates microbial growth and the lowest concentration at which no growth occurs is the MIC.

### 3.7. Determination of Antioxidant Activity

#### 3.7.1. Scavenging Activity of DPPH Radical

The antioxidant activity was assessed using the DPPH free radical scavenging test as described previously by Brand-Williams et al. [34]. This method measures the anti-radical activity of the free radical DPPH of the investigated oil. DPPH is a violet stable free radical molecule, which can be reduced by the presence of an antioxidative sample to yellow DPPH. Various concentrations (10, 50, 100, 500, and 1000 μg/mL) of the volatile oil were tested. The test mixture contained, in a total volume of 1 mL, 500 μL of the oil, 125 μL prepared DPPH (1 mM in methanol) and 375 μL methanol. Then, the mixture was incubated for 30 min in the dark, at room temperature. Finally, the absorbance was recorded at λ = 517 nm and the anti-radical activity was calculated from the following equation:

% of anti-radical activity = Abscontrol − Abssample/Abscontrol × 100

#### 3.7.2. β-Carotene-Linoleic Acid Assay

To investigate the antioxidant activity of the essential oil of *L. inflata*, the β-carotene bleaching assay was used according to Mohd-Esa et al. [35]. β-carotene solution (0.2 mg/mL) was dissolved in chloroform and then 1 mL of this solution was added to a solution of 0.2 mL of Tween-20 and 0.02 mL of linoleic acid. After removing the chloroform, the mixture was diluted with 100 mL of distilled water and mixed for 2 min. A volume of 0.2 mL of the oil (1 mg/mL) was added to 5 mL of the mixture and incubated at 40 °C for 2 h. The optical density was measured at 470 nm at 15 min intervals using a UV-visible spectrophotometer (UV mini-1240, Shimadzu, Japan). Rutin was utilized as a positive control (1 mg/mL). The antioxidant activity was determined using the following equation:

Antioxidant activity (%) = (Abs0 − Abst)/(Abs°0 − Abs°t) × 100

where, Abs0 and Abs°0 are the optical density values determined at zero time of incubation for the oil and the control, respectively. Abst and Abs°t are the optical density values for the oil and the control, respectively, at 120 min.

## 4. Conclusions

In the present work, we reported, for the first time, the chemical composition of the volatile oil of *L. inflata* and its antimicrobial and antioxidant effects. GC and GC/MS investigations indicated that the chemical composition was identified by a high content of aliphatic acids (51.1%), where hexadecanoic acid (32.8%) and *n*-dodecanoic acid (7.8%) were the fundamental components. Camphor (6.1%) and linalool (3.2%) were detected as essential compounds among the oxygenated monoterpenoids. The GC investigation of the oil disclosed that the chemical content basically varied from that of other investigated African and Indian *Leucas* members. The results obviously indicated that the volatile oil has an interesting antibacterial, but a moderate antioxidant, activity. Consequently, our results bolster the suggestion that *L. inflata* can be a promising source of antibacterial agents. Further work is required to isolate the unidentified components from the oil, as well as to ensure antimicrobial activity on other bacterial strains.

## Figures and Tables

**Table 1:** Chemical composition of the essential oil of *Leucas inflata*.

No.	Compounds	RI	% Occurrence	Identification
1	α-Pinene	932	0.5	1, 2, 3
2	1-Heptene-3-ol	954	0.6	1, 2
3	1-Octene-3-ol	961	0.9	1, 2
4	2-Pentylfuran	980	0.5	1, 2
5	trans-Linalool oxide	1059	0.3	1, 2
6	Fenchone	1070	1.1	1, 2, 3
7	Linalool	1084	3.2	1, 2, 3
8	α-Fenchol	1101	2.3	1, 2, 3
9	Exo-Fenchol	1109	0.2	1, 2, 3
10	Camphor	1124	6.1	1, 2, 3
11	Borneol	1152	0.3	1, 2, 3
12	Terpinen-4-ol	1165	0.5	1, 2, 3
13	α-Terpineol	1175	1.1	1, 2
14	Myrtenol	1182	0.4	1, 2
15	*t*-Geraniol	1236	0.4	1, 2
16	Nonanoic acid	1257	1.2	1, 2
17	Thymol	1272	0.6	1, 2, 3
18	Carvacrol	1285	0.3	1, 2, 3
19	Eugenol	1333	0.2	1, 2, 3
20	Decanoic acid	1353	0.7	1, 2
21	(*E*)-β-Damascenone	1365	0.4	1, 2
22	Methyleugenol	1374	0.2	1, 2
23	Dodecanal	1388	0.7	1, 2
24	(*E*)-β-Caryophyllene	1423	1	1, 2, 3
25	Undecanoic acid	1450	1.3	1, 2
26	α-Humulene	1456	0.5	1, 2
27	Alloaromadenderene	1461	0.2	1, 2
28	γ-Muurolene	1474	0.8	1, 2
29	β-Selinene	1487	0.7	1, 2
30	α-Muurolene	1497	0.6	1, 2
31	7-epi-α-Selinene	1519	0.8	1, 2
32	*n*-Dodecanoic acid	1552	7.8	1, 2
33	Caryophyllene oxide	1579	1.7	1, 2, 3
34	Humulene epoxide II	1604	0.7	1, 2
35	*t*-Cadinol	1633	1.5	1, 2
36	β-Eudesmol	1646	2	1, 2
37	Eudesm-11-en-4α-ol	1653	0.8	1, 2
38	*n*-Tetradecanoic acid	1744	3.9	1, 2
39	6,10,14-Trimethylpentadecane-2-one	1828	6.3	1, 2
40	Pentadecanoic acid	1841	0.5	1, 2
41	Hexadecanoic acid	1953	32.8	1, 2
42	Phytol	2102	0.5	1, 2
43	(*Z*,*Z*)-9,12-Octadecadienoic acid	2120	4.4	1, 2
	Monoterpene hydrocarbons		2.5	
	Oxygenated monoterpenes		16	
	Sesquiterpene hydrocarbons		5	
	Oxygenated sesquiterpenes		6.7	
	Aliphatic acids		51.1	
	Other compounds		7.9	
	Total		89.2	

RI, retention indices relative to C8-C30 n-alkanes on the CP-Sil 5 CB column; tr: traces, 1: retention index; 2: mass spectrum; 3: co-injection with authentic compound.

**Table 2:** Antimicrobial activity (minimal inhibitory concentrations (MIC-values)) of the essential oil of *L. inflata*.

Plant Species	MIC a				
	*S. aureus*	*B. subtilis*	*E. coli*	*P. aeruginosa*	*C. albicans*
*L. inflata*	0.81	0.81	-	-	-
Amoxicillin	3.5	3.5	nt	nt	nt
Gentamicin	nt	nt	3.5	7	nt
Nystatin	nt	nt	nt	nt	3.5

^a^: minimum inhibitory concentration values are given as mg/mL for essential oils and μg/mL for standard antibiotics. nt: not tested.

**Table 3:** Free radical scavenging activity and antioxidant activity of *L. inflata* essential oil.

Sanmples	Radical Scavenging Activity in (%)					Total Antioxidant Activity (%)
	10	50	100	500	1000	1000
	(µg/mL)					(µg/mL)
*L. inflata*	6.9	12.7	20.8	28.7	38.1	31.8
Ascorbic acid	48.2	80.5	91.8	96.1	96	nt
Rutin	nt	nt	nt	nt	nt	90.9

nt: not tested.
